# Validation of the Persian version of dysphagia in multiple sclerosis questionnaire for the assessment of dysphagia in multiple sclerosis

**Published:** 2018-07-06

**Authors:** Zahra Jafari, Mohammad Rahim Shahbodaghi, Saman Maroufizadeh, Abdorreza Naser Moghadasi

**Affiliations:** 1 Department of Speech Therapy, School of Rehabilitation, Tehran University of Medical Sciences, Tehran, Iran; 2 Department of Epidemiology and Reproductive Health, Reproductive Epidemiology Research Center, Royan Institute for Reproductive Biomedicine, Academic Center for Education, Culture and Research, Tehran, Iran; 3 Multiple Sclerosis Research Center, Neuroscience Institute, Tehran University of Medical Sciences, Tehran, Iran

**Keywords:** Dysphagia, Multiple Sclerosis, Reliability and Validity, Dysphagia in Multiple Sclerosis, Deglutition Disorders

## Abstract

**Background:** About one third of patients with multiple sclerosis (MS) experience dysphagia. Dysphagia can cause complications such as malnutrition, lung infections, aspiration pneumonia and dehydration, thus it is very helpful to diagnose dysphagia as soon as possible. The aim in this study was to translate dysphagia in multiple sclerosis (DYMUS) questionnaire into Farsi and and validate it.

**Methods:** Forward-backward method was used to translate original English version of DYMUS into farsi, and then the questionnaire (one for each patient) was filled in through asking questions from 200 patients with MS by a speech and language pathologist. Confirmatory factor analysis (CFA) was used to examine the construct validity. Internal consistency and test-retest reliability of the DYMUS were evaluated using Cronbach’s alpha and the intraclass correlation coefficient (ICC), respectively.

**Results:** CFA showed that a two-factor model of DYMUS including “dysphagia for solid” and “dysphagia for liquid” fitted the data well [relative chi-square [χ^2^/degree of freedom (df) = 1.55, CFI = 0.967, Tucker-Lewis index (TLI) = 0.957, root mean square error of approximation (RMSEA) = 0.053, and weighted root mean square residual (WRMR) = 0.874]. The Cronbach’s alpha for total score and liquid and solid subscales were 0.776, 0.557, and 0.725, respectively. The DYMUS showed adequate test-retest reliability for the total and subscales (ICC: 0.880-0.956). Moreover, this study has shown a significant relationship between DYMUS score and Expanded Disability Status Scale (EDSS) score, disease duration, type of MS, and self-reported dysphagia.

**Conclusion:** The Persian version of DYMUS is a reliable and valid tool to screen dysphagia among patients with MS.

## Introduction

Dysphagia is difficulty in moving food from mouth to stomach and can be caused by a combination of impairments of various structures such as cerebellar, brainstem corticobulbar tracks and lower cranial nerves.^1^

Multiple sclerosis (MS) is a demyelinating disease in which the immune system of body attacks the central nervous system (CNS) and multiple plaques appear in patient’s nervous system. Because of the wide variation in CNS involvement in MS, there is potential for abnormality in almost every aspect of swallowing physiology in patients with MS.^2^ Dysphagia is a common symptom amongpatients with MS and occurs in 33-43% of them.^3^^-^^5^ In patients with MS, dysphagia is caused by neurological lesions that occurs in cortex, cerebellar, brainstem, or cranial nerves and its symptoms increase with disease progression and increased deficiency in cerebellar and brainstem parts.^1^ Dysphagia decreases quality of life (QOL) of patients with MS and can cause severe problems such as dehydration and aspiration pneumonia and is a source of life threating complications.^5^ With the increase of disability, the incidence rate of dysphagia increases, as reaching 65% in more severe cases.^5^^-^^7^ Dehydration and aspiration pneumonia are of the common causes of death in final stages of MS. Since dysphagia can cause severe complications and sometimes patients themselves or their caregivers underestimate dysphagia symptoms, there is a need for a reliable tool for screening swallowing problems among patients with MS and identifying patients who are at the risk of dysphagia needing specific instrumental investigations. In addition, if needed, a rehabilitation program should be started to prevent or reduce its complications.^8^^,^^9^

There is no specific questionnaire for the assessment of dysphagia in MS except DYMUS, which was developed in 2008 by Bergamaschi, et al. to identify the risk of dysphagia in patients with MS.^10^ DYMUS questionnaire is an non-invasive instrument for screening dysphagia in patients with MS, and takes the advantages of easy administration and taking a short period of time to implement and in contrast to instrumental evaluations which are mostly not accessible, it can easily be available. Bergamaschi, et al. validated DYMUS among 1875 patients with MS in 2010.^9^ The questionnaire is translated and validated in French and Portuguese and the results were indicative of the satisfactory psychometric properties of DYMUS.^3^^,^^11^

The presented study was accomplished with the aim to translate DYMUS to Persian and investigate its validity and reliability in order for screening of dysphagia in MS.

## Materials and Methods

This cross sectional study was performed on 200 patients with MS who were visited at the MS clinic of Sina Hospital, Tehran, Iran. Patients with a history of other demyelinating diseases or diseases which could cause dysphagia such as cerebrovascular accident (CVA) were excluded from the study.

***Questionnaires***

*Dysphagia in MS (DYMUS):* A committee of Italian neurologists developed DYMUS questionnaire which was the first questionnaire developed to screen dysphagia in patients with MS.^8^ The DYMUS was a self-report questionnaire which consisted of 10 questions and two subscales: dysphagia for solid (positive scores in questions 1, 3, 4, 5, 7, 8, or 10) and dysphagia for liquids (positive scores to questions 2, 6, or 9). Answers were coded as 0 (negative answers) or 1 (positive answers), depending on the absence or presence of the event. The scores ranged from 0 to 10 and dysphagia was identified by at least one positive response to questions (score = 1) and it was considered alarming when the score was equal to or more than 3 (score ≥ 3).

*Demographic characteristics:* The patients were asked to indicate their sex, age, and duration of disease and a neurologist indicated patient’s Expanded Disability Status Scale (EDSS) and type of MS. All patients were asked to express either they had problems in swallowing or not and then, without consideration of their answers, a speech and language pathologist administrated the questionnaire.

Firstly, permission was received to translate the DYMUS to Persian from Bergamaschi, et al. who have developed the questionnaire. Then, the reeserachers translated DYMUS according to the standard “forward-backward” translation protocol. Firstly, two Persian translators who were expert and fluent in English, translated the original version of DYMUS questionnaire into Farsi independently. Then, the two forward translations were compared and synthesized into one common version by the research team. This forward version was given to two translators for translating the questionnaire back into English. The two backward translations were reviewed by experts in the field of dysphagia and MS (speech and language pathologists and neurologists) and were compared and variances were corrected. 20 patients with MS were asked to answer the provisional Persian version of DYMUS as a pilot study. Further corrections were conducted and the final original version of the questionnaire was made. In order for conceptual equivalence with the original source version, the final English version was sent to the developer of questionnaire (Bergamaschi) and he confirmed that it was equivalent to the original version.

Confirmatory factor analysis (CFA) was used to investigate the original two-factor structure of the DYMUS questionnaire proposed by Bergamaschi, et al.^10^ The weighted least squares mean and variance adjusted (WLSMV) estimation method was used in CFA due to dichotomous item scores. The goodness of fit was assessed using the chi-square, relative chi-square [χ^2^/degree of freedom (df)], the root mean square error of approximation (RMSEA), Tucker-Lewis index (TLI), the comparative fit index (CFI), and the weighted root mean square residual (WRMR). An insignificant chi-square indicated a good model fit with the data, but it was sensitive to sample size. An alternate evaluation of the χ^2^ value was to examine the ratio of χ^2^ to the degrees of freedom for the model (relative chi-square). A χ^2^/df ratio of less than 2 was considered to be indicative of a good fit between the hypothetical model and the sample data. In addition, cut-off values of above 0.95, below 0.06, and below 0.9 for respectively TLI and CFI, RMSEA, and WRMR were indicative of a relatively good fit between the hypothesized model and the observed data.^12^^-^^14^ Intraclass correlation coefficient (ICC) and Cronbach’s alpha were used to evaluate the test-retest reliability and internal consistency of the DYMUS . A Cronbach’s α of above 0.80, above 0.70, and 0.60-0.70 were considered excellent, satisfactory, and acceptable, respectively. Moreover, an ICC of above 0.75 indicated the excellent test-retest reliability.^15^^,^^16^ Furthermore, Spearman's correlation coefficient, Kruskal-Wallis test (followed by post-hoc Dunn’s test), and Mann-Whitney test were exploited to examine the relationship between DYMUS scores and demographic characteristics. 

All data were analyzed using the SPSS software (version 17, SPSS Inc., Chicago, IL, USA), except for the CFA, which was conducted using Mplus software version 6.12 (Muthén & Muthén, Los Angeles,CA, USA). All statistical tests were 2-sided and a P value of less than 0.05 was considered to be statistically significant.

## Results

***Characteristics of patients: ***In total, 200 patients with MS (32 men and 168 women) were included in the study. The mean age of the patients was 34.54 ± 8.53 years in the age range of 20-60 years old and the mean duration of the disease was 5.95 ± 4.56 years in the range of 1-25 years. Of the patients, 151 (75.5%), 41 (20.5%), and 8 (4.0%) had relapsing-remitting MS (RRMS), secondary-progressive MS (SPMS), and primary-progressive MS (PPMS), respectively. In addition, 27 (13.5%) of the patients reported suffering from dysphagia before answering the questionnaire by themselves. The mean EDSS score was 5.95 ± 4.56 in the range of 0.0-7.5.

Table1 displays the percentages of positive answers to the DYMUS items and prevalence of dysphagia. 

**Table 1:** Percentages of positive responses to the dysphagia in multiple sclerosis (DYMUS) items and prevalence of dysphagia

**Item**	**n (%)**
Do you have difficulties swallowing solid food (such as meat, bread, and the like)?	23 (11.5)
Do you have difficulties swallowing liquid (such as water, milk, and the like)?	19 (9.5)
Do you have a globus sensation in your throat during swallowing?	24 (12.0)
Do you have food sticking in your throat?	26 (13.0)
Do you cough or do you have a choking sensation after solid ingestion?	24 (12.0)
Do you cough or do you have a choking sensation after liquid ingestion?	24 (12.0)
Do you need to swallow more and more times before completely swallowing solid food?	24 (12.0)
Do you need to cut food in small pieces before swallowing?	25 (12.5)
Do you need to take more and more sips before completely swallowing liquid?	31 (15.5)
Do you have weight loss?	29 (14.5)
Dysphagia for solid	78 (39.0)
Dysphagia for liquid	52 (26.0)
Dysphagia for both solid and liquid	45 (22.5)
Dysphagia	85 (42.5)
Alarming dysphagia	42 (21.0)

**Table 2:** The results of confirmatory factor analysis (CFA) of two-factor model of dysphagia in multiple sclerosis (DYMUS)

**Item**	**Solid**	**Liquid**
Do you have difficulties swallowing solid food (such as meat, bread, and the like)?	0.93	
Do you have a globus sensation in your throat during swallowing?	0.63	
Do you have food sticking in your throat?	0.55	
Do you cough or do you have a choking sensation after solid ingestion?	0.75	
Do you need to swallow more and more times before completely swallowing solid food?	0.88	
Do you need to cut food in small pieces before swallowing?	0.75	
Do you have weight loss?	0.61	
Do you have difficulties swallowing liquid (such as water, milk, and the like)?		0.82
Do you cough or do you have a choking sensation after liquid ingestion?		0.66
Do you need to take more and more sips before completely swallowing liquid?		0.79

The mean DYMUS score was 1.25 ± 1.90 with the range of 0-10. The lowest and highest positive responses to the DYMUS items were observed for item 2 and item 9, respectively. According to DYMUS, 42.5%, 39.0%, 26.0%, and 22.5% of the patients had dysphagia, dysphagia for solid, dysphagia for liquid, and dysphagia for simultaneously solid and liquid, respectively. 27 (13.5%) patients declared that they had problems in swallowing at the time of interview, and after answering the DYMUS questionnaire, the results revealed that 92.6% of them had swallowing problems and 81.5% among them had alarming dysphagia. Among patients who reported no difficulty in swallowing before answering DYMUS questionnaire (173 patients), 34.0% and 11.6% had swallowing problems and alarming dysphagia, respectively.

***CFA:*** The CFA was used to evaluate the goodness of fit of the two-factor model of DYMUS. The fit indices indicated a good fit of the data to the model (χ^2^ = 52.88, df = 34, P = 0.020; χ^2^/df = 1.55; TLI = 0.957; CFI = 0.967; WRMR = 0.874 and RMSEA = 0.053). As presented in table 2, all standardized factor loadings were significant and in the expected direction, ranging from 0.55 to 0.93.

***Reliability analysis:*** Cronbach’s alpha coefficients for assessing internal consistency of the DYMUS were as follows: Solid subscale (7 items, 0.725), liquid subscale (3 items, 0.557), and total score (10 items, 0.776). The two-week test-retest reliability of the DYMUS among 40 patients using ICC was 0.944, 0.956, and 0.880 for the total score, solid subscale, and liquid subscale, respectively.

***Relationship between DYMUS and demographic characteristics:*** Given the correlation coefficients, the total DYMUS score had significantly positive correlation with disease duration (r = 0.184, P = 0.009) and EDSS (r = 0.583, P < 0.001). Regarding the type of MS, the total DYMUS score was lower among patients with RRMS compared to other patients (P < 0.001). 

**Table 3:** Relationship between dysphagia in multiple sclerosis (DYMUS) and demographic/clinical characteristics in patients with MS

**Variable**	**Solid**		**Liquid**		**Total**	
	**r or Mean ± SD**	**P**	**r or Mean ± SD**	**P**	**r or Mean ± SD**	**P**
Age	0.084	0.238	0.055	0.439	0.109	0.125
Duration	0.139	0.049	0.169	0.017	0.184	0.009
EDSS	0.562	< 0.001	0.482	< 0.001	0.583	< 0.001
Sex		0.561		0.537		0.512
Men	0.69 ± 1.09		0.28 ± 0.58		0.97 ± 1.47	
Women	0.91 ± 1.48		0.39 ± 0.74		1.30 ± 1.97	
Type of MS		< 0.001		< 0.001		< 0.001
RRMS	0.59 ± 1.20		0.26 ± 0.67		0.85 ± 1.62	
SPMS	1.80 ± 1.72		0.73 ± 0.78		2.54 ± 2.19	
PPMS	1.50 ± 1.69		0.50 ± 0.76		2.00 ± 2.39	
Self-reported dysphagia		< 0.001		< 0.001		< 0.001
Yes	3.11 ± 1.91		1.44 ± 1.01		4.56 ± 2.29	
No	0.53 ± 0.94		0.20 ± 0.48		0.73 ± 1.19	

EDSS: Expanded Disability Status Scale; RRMS: Relapsing-remitting MS; SPMS: Secondary-progressive MS; PPMS: Primary-progressive MS; SD: Standard deviation

Moreover, patients who reported dysphagia (4.56 ± 2.29) scored significantly higher than other patients (0.73 ± 1.19) on total DYMUS (P < 0.001). As presented in table 2, there was no significant relationship between total DYMUS and age (P = 0.125) and sex (P = 0.512). Moreover, as seen in table 3, the same results were obtained for solid and liquid subscales.

## Discussion

The present study was accomplished aiming to investigate the reliability and validity of the DYMUS among the Iranian patients with MS. To our knowledge, this is the first study evaluating the factor structure of DYMUS by CFA. The two-factor model of DYMUS proposed by Bergamaschi, et al.^10^ was tested and it was found that a two-factor model fit the data well and all standardized factor loadings were significant. 

The DYMUS was found to have high internal consistency (α = 0.776) indicating that it is a reliable instrument for measuring dysphagia in MS. This finding is quite consistent with previous studies that have reported high internal consistency for DYMUS questionnaire.^1^^,^^9^^,^^10^ Moreover, the test-retest reliability of DYMUS, two weeks after initial testing was ICC = 0.944, indicating good stability over time.

In the present study, there was a correlation between DYMUS scores and types of MS, EDSS scores, duration of disease, and patients self-report of dysphagia. Among the clinical types of the disease, PPMS and SPMA were more associated with alarming dysphagia while RRMS presented milder dysphagia. Regarding the EDSS, higher scores were associated with the high incidence rate of dysphagia. Patients with a longer duration of disease had higher DYMUS scores and patients who reported dysphagia had significantly higher DYMUS scores and all of these finding are in line with previous similar studies.^3^^,^^17^ As expected, no significant relation was found between DYMUS scores and sex.

The prevalence of dysphagia which was measured by DYMUS in the original study of DYMUS carried out by Bergamaschi, et al.^10^^,^^18^ and in another validation study of DYMUS conducted by them, was 35% and 31%, respectively. In the studies by Villain and Heinzlef^11^ and Sales, et al.,^3^ percentage of at least one abnormal answer to DYMUS which shows dysphagia, was 23% and 58%. In the current study, the incidence rate of dysphagia was 42.5% and DYMUS showed dysphagia 3.14 times more than patients’ self-report of dysphagia, so it indicates the importance of using a screening tool such as DYMUS to identify dysphagia among patients with MS and submit them for instrumental evaluation of swallowing including time barium swallow and video fluoroscopy for them and starting rehabilitation programs in order to prevent complication.

Some limitations were along with this study. First, the main limitation of this study was that it was performed in a single center, however the main results can be applied to other centers as well. Secondly, this is a cross-sectional study and causal relationship between dysphagia and demographic/clinical characteristics of patients with MS cannot be established. Thirdly, to evaluate the sensitivity and specificity of DYMUS questionnaire, it should be tested versus instrumental tests such as videofluoroscopy or fiberoptic endoscopic evaluation of swallowing (FEES) and the correlation between DYMUS scores and instrumental tools assessment data should be analyzed.

## Conclusion

The present study showed that the Persian version of DYMUS is a valid and reliable tool and it can be used for early screening of dysphagia in patient with MS.

## Notes:

**How to cite this article:** Jafari Z, Shahbodaghi MR, Maroufizadeh S, Naser-Moghadasi A. Validation of the Persian version of dysphagia in multiple sclerosis questionnaire for the assessment of dysphagia in multiple sclerosis. Iran J Neurol 2018; 17(3): 99-104.
